# Visualization and Heat Transfer Performance of Mini-Grooved Flat Heat Pipe Filled with Different Working Fluids

**DOI:** 10.3390/mi13081341

**Published:** 2022-08-18

**Authors:** Fei Xin, Qiang Lyu, Wenchao Tian

**Affiliations:** 1 Key Laboratory of Ministry of Education for Electronic Equipment Structure Design, Xidian University, Xi’an 710071, China; 2 State Key Laboratory of Multiphase Flow in Power Engineering, Xi’an Jiaotong University, Xi’an 710049, China

**Keywords:** mini-grooved flat heat pipe (MGFHP), visualization, heat transfer, working fluid, filling ratio, wettability

## Abstract

Mini-grooved flat heat pipe (MGFHP) possesses the advantages of high compactness, no mechanical component, super thermal conductivity, and excellent temperature uniformity, which can meet the demand for electronic devices efficiently cooling. In this research, visual and heat transfer experiments were performed to investigate the flow and thermal characteristics inside the MGFHP. Fluid flow and distribution are observed to be quite different in the MGFHP containing different working fluids, which is affected by the physical properties of working fluid, the surface state of the grooved wick, and limited working space. Additionally, the input heat, working fluid type, filling ratio, and wettability obviously affect the thermal conductivity and temperature uniformity of the MGFHP. The deionized water-filled MGFHP possesses lower thermal resistance and higher heat transfer capacity than anhydrous ethanol or hexane filled MGFHP, especially for the copper oxide MGFHP filled with deionized water with a filling ratio of 1.0. Thermal resistance, maximum temperature, and temperature nonuniformity at the condensation section of deionized water-filled copper oxide MGFHP are lower than those of the original copper MGFHP by 31.1%, 3.7 °C, and 0.11 °C for the anhydrous ethanol filled MGFHP and 34.4%, 3.1 °C, and 0.13 °C for the hexane filled MGFHP, respectively.

## 1. Introduction

Nowadays, with the development of advanced interconnection and assembly technologies for electronic packaging, the thermal stress problem has become one of the most important challenges for electronic devices [1,2]. In general, the normal working temperature of electronic devices is in the range of −5–65 °C under the steady state. However, the reliability of chips decreases sharply by 5% for every 1 °C increase in chips surface temperature when the working temperature exceeds 70–80 °C [3]. Moreover, the uneven temperature distribution may cause large thermal stress inside the electronic devices, which affects the working stability of the electronic system significantly [4]. Employing an efficient and reliable approach to remove the considerable amount of heat from the electronics within a small space is imperative [5]. Mini flat heat pipe, as one kind of the vapor-liquid phase-change device, has the advantages of compact structure, no additional electric drive, large thermal conductivity, and uniform temperature distribution, which can meet the requirement of cooling electronics effectively [6,7].

**Figure 1:**
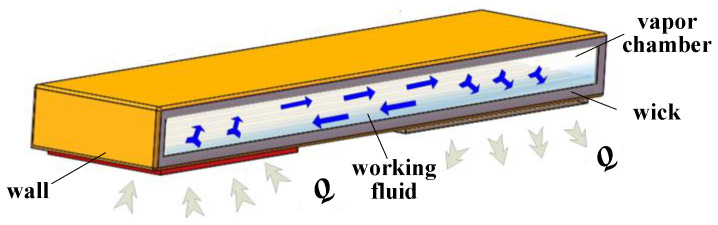
Structure of mini flat heat pipe.

Mini flat heat pipe basically comprises wall, wick, vapor chamber, and working fluid, as presented in Figure 1. As the main thermal resistance exists in the wick of the heat pipe [8,9], there are two methods to improve the thermal performance of the heat pipe. One is optimizing the wick structure, and the other is choosing the suitable working fluid [10,11]. On account of grooved wick integrated with the wall possessing comparatively low thermal flow resistance, mini-groove can be used as wick for the heat pipe to cool electronic devices [12,13]. Meanwhile, as the mini-grooved flat heat pipe (MGFHP) chiefly employs the phase change in working fluid to deliver substantial heat, the option of working fluid type is crucial to the performance of the MGFHP. Numerous factors need to be considered to choose the working fluid for the MGFHP, such as the compatibility of working fluid with the wick, working temperature, cost, toxicity, heat transfer capacity of working fluid, and so on. Various working fluids have been used in the MGFHP for cooling electronics. Lips et al. [14] conducted an experiment to study the thermal behavior of a grooved flat heat pipe filled with methanol. They paid special attention to the effect of boiling in the heat pipe, finding that the existence of nucleate boiling in the grooves improved the heat transfer performance of the heat pipe. Wang et al. [15] used deionized water filled in the flat heat pipes with interlaced grooves to experimentally investigate the performance of the heat pipe for CPU cooling. The heat transfer characteristics of the heat pipe were studied in both axial and radial directions. Chen and Chou [16] explored the influence of liquid filling ratios and leakage on the cooling effect of Al 6061 flat heat pipe filled with acetone. They found that the heat pipe filled with 25% amount of working fluid performed optimally with high heat transfer limit and thermal conductivity. Wang et al. [17] experimentally investigated the thermal performance of flat heat pipe containing different working fluids (methanol, acetone, and R141b). They found that the methanol-filled heat pipe possessed lower thermal resistance.

However, the distribution of different working fluids inside the heat pipe was unclear in the above literature, and the influences of working fluid properties and wettability on the thermal characteristics of the flat heat pipe need to be further studied. In this way, visual and thermal experiments were conducted on the copper-based mini-grooved flat heat pipe with different working fluids, aiming to clearly understand the effects of working fluid properties on the flow and thermal characteristics of the MGFHP. DI water, anhydrous ethanol, and hexane are common, economical, and relatively environment-friendly fluids. They are compatible with copper-based MGFHP, and their working temperatures are suitable for the cooling of electronic devices. Thus, these three typical kinds of fluids were chosen to experimentally explore the flow and thermal performance of the MGFHP. The influences of input heat, working fluid type, filling amount, and surface state of the grooved wick on the MGFHP were also examined.

## 2. Visual and Heat Transfer Experiments Set-Up

**Figure 2:**
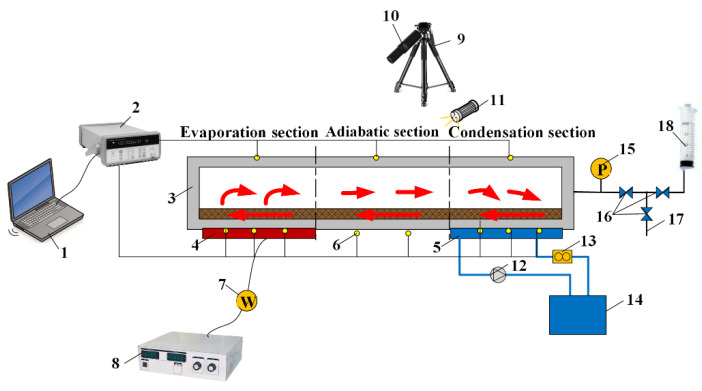
System diagram of visual and heat transfer experiments: 1-computer, 2-data collection, 3-MGFHP, 4-heat chip, 5-water jacket, 6-thermocouple, 7-watt meter, 8-power supply, 9-CCD camera, 10-microscope, 11-light source, 12-water pump, 13-flowmeter, 14-cold water storage, 15-pressure meter, 16-vacuum valve, 17-vent, and 18-injector.

**Figure 3:**
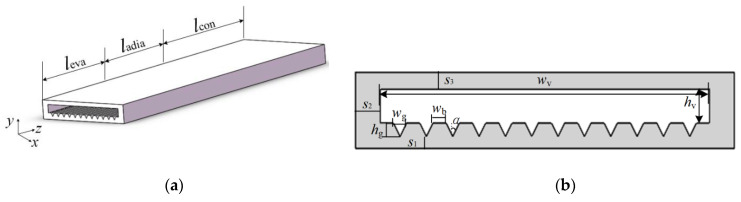
Geometric structure of the MGFHP: (**a**) 3D structure; (**b**) Cross-section in the *z*-direction.

The system diagram of the visual and heat transfer experiments for the copper-based MGFHP is presented in Figure 2. The experimental system was composed of heat source, heat sink, measurement and data collection, vacuum pumping, and liquid filling parts [18,19]. The MGFHP was divided into evaporation, adiabatic, and condensation sections. Constant heat flux provided by a ceramic heat chip was applied at the evaporation section to offer the heat source. Water jacket with an inlet water flow rate of 400 mL·min^−1^ and a temperature of 20 °C was used as the heat sink at the condensation section. The heat source area was 20.0 × 20.0 mm^2^, and the heat sink area was 40.0 × 20.0 mm^2^. V-type groove was chosen as the wick for the MGFHP, located at the lower copper base. It was produced by laser engraving. The structure and geometric parameters of the MGFHP are shown in Figure 3 and Table 1, respectively. The lengths of evaporation, adiabatic, and condensation sections were separately 20.0 mm, 20.0 mm, and 40.0 mm.

Because the wall material could affect the thermal performance of the MGFHP, the material of the MGFHP upper cover adopted copper or glass [20]. Copper was selected as the upper cover material for the thermal experiment to measure the heat transfer performance of the MGFHP. Upper glass cover and lower copper base were bonded by diffusion bonding under the welding temperature of 850 °C. Additionally, the MGFHP was wrapped with insulation cotton in the thermal experiment, with the purpose of reducing the heat leakage. As for the visual experiment, Pyrex 7740 glass was used as the upper cover material of the MGFHP to observe the fluid flow and distribution inside the MGFHP [21]. It was glued with the lower copper base. Additionally, CCD camera with an optical microscope was utilized to visualize the fluid flow and liquid-vapor distribution inside the MGFHP. 

The MGFHP was required to evacuate the air and charge the working fluid before utilizing. A vacuum pump was utilized to degas the MGFHP until the pressure inside the MGFHP was sustained below 1 Pa for 1 h. Additionally, an injector with an accuracy of ±0.01 mL was utilized to fill the working fluid into MGFHP. Deionized water, anhydrous ethanol, or hexane was filled into the MGFHP as working fluid. The ratio of working fluid filling volume $V_{f}$ to grooves volume $V_{g}$ is used to evaluate the filling amount of working fluid inside the MGFHP:

(1) $$\eta =V_{f}/V_{g}$$

**Figure 4:**
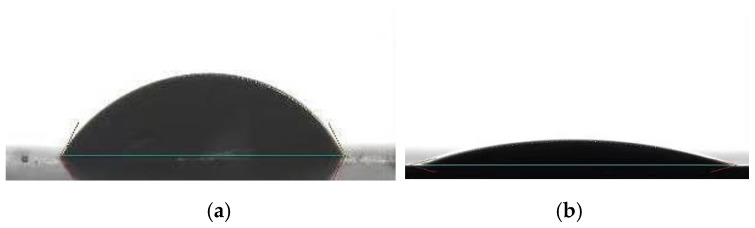
Contact angles of surfaces with deionized water: (**a**) copper surface; (**b**) copper oxide surface.

A contact angle measuring instrument with the uncertainty of ±0.1° was utilized to measure the contact angle between the copper and working fluid. Each condition was measured three times, and the average value was taken. The wettability of deionized water with copper was measured and found to be comparatively poor with the contact angle of 63.7°, as shown in Figure 4a. A method was taken to oxidize the copper surface to construct a CuO film on the surface, which could improve the surface energy and wettability. Specifically, one side of the copper was coated with ink to prevent oxidation, while the other side was bared to be oxidized. The copper was immersed into a mixture solution with 40 mL 10.0 mol·L^−1^ NaOH, 20 mL 1.0 mol·L^−1^ (NH_4_)_2_S_2_O_8_, and 90 mL water for 1 h under 20 °C and then was dried in the drying oven for 3 h under 180 °C. The mechanism of copper surface oxidation [22] is as follows:

(2) $${\mathrm{Cu}+4\mathrm{NaOH}+(\mathrm{NH}}_{4}{)}_{2}S_{2}O_{8}\to {\mathrm{Cu}(\mathrm{OH})}_{2}{+2\mathrm{Na}}_{2}{\mathrm{SO}}_{4}{+2\mathrm{NH}}_{3}↑{+2H}_{2}O$$

(3) $${\mathrm{Cu}(\mathrm{OH})}_{2}\to {\mathrm{CuO}+H}_{2}O$$

The contact angle of deionized water with copper oxide was measured to be 13.5°, as shown in Figure 4b. Meanwhile, the measured contact angles of anhydrous ethanol and hexane with copper were 12.8° and 10.3°, respectively. Anhydrous ethanol and hexane present better wettability with copper.

**Figure 5:**
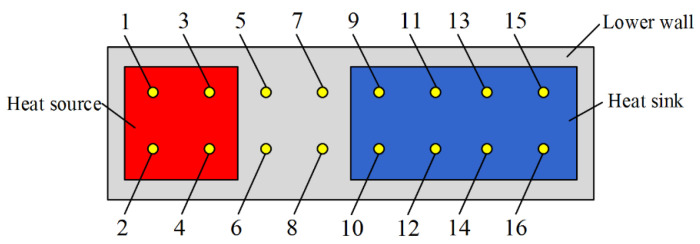
Distribution of thermocouples 1–16 at the lower surface of the MGFHP.

Calibrated thermocouples with the uncertainty of ±0.1 °C were applied to measure the temperature of the MGFHP. The location and function of calibrated thermocouples utilized in the experiment are shown in Table 2. Figure 5 presents the distribution of calibrated thermocouples 1–16 at the lower surface of the MGFHP. The experiment time for every case was 1800 s. Experiments were repeated three times for every case, and the average values were taken.

Thermal resistance, maximum temperature at the evaporation section, and temperature nonuniformity at the condensation section are adopted to characterize the thermal performance of the MGFHP [15]. The definitions of these evaluation parameters are as follows:

(1) Heat transfer amount $Q^{\prime }$:

Although insulation cotton is wrapped around the MGFHP to preserve heat, a small amount of heat is still released from the MGFHP surface to the outside in the form of natural convection. It mainly happens on the upper and lower surface of the MGFHP at the evaporation and adiabatic sections. The heat transfer amount is the input heat amount minuses heat loss [23]:

(4) $$Q^{\prime }=Q-Q_{\mathrm{loss}}$$

(2) Thermal resistance R:

(5) $$R=({\bar{T}}_{\mathrm{eva}}-{\bar{T}}_{\mathrm{con}})/Q$$

where ${\bar{T}}_{\mathrm{eva}}$ and ${\bar{T}}_{\mathrm{con}}$ are average temperatures at heat source and heat sink areas, respectively. In the experiment, they are defined as follows:

At the evaporation section with heat source:

(6) $${\bar{T}}_{\mathrm{eva}}=(T_{1}+T_{2}+T_{3}+T_{4})/4$$

At the condensation section with heat sink:

(7) $${\bar{T}}_{\mathrm{con}}=(T_{9}+T_{10}+\ldots T_{16})/8$$

(3) Temperature nonuniformity at the condensation section $dT_{\mathrm{con}}$ [24]:

(8) $$dT_{\mathrm{con}}=\sqrt{[\sum_{i=1}^{8}{\left(T_{\mathrm{con},i}-{\bar{T}}_{\mathrm{con}}\right)}^{2}]/8}$$

where $T_{\mathrm{con},i}$ is the temperature of calibrated thermocouples 9–16 at the condensation section.

The experiment correlation of large space natural convection with uniform wall temperature boundary condition is employed to calculate the heat loss in the experiment. The physical properties of air are chosen under the average temperature $T_{\mathrm{ave}}$ of wall surface temperature $T_{w}$ and environment temperature $T_{17}$:

(9) $$T_{\mathrm{ave}}=(T_{w}+T_{17})/2$$

According to the boundary condition of uniform wall temperature, the correlation of fluid under the large space natural convection is:

(10) $$Nu_{\mathrm{ave}}=C{\left(Gr\times Pr\right)}^{n}$$

Grashof number is:

(11) $$Gr=\frac{\rho^{2}g\alpha^{*}l_{\mathrm{ave}}{}^{3}\Delta T}{\mu^{2}}$$

where $l_{\mathrm{ave}}$ is the feature length, which is the measured surface length. ΔT is the difference between wall surface temperature $T_{w}$ and environment temperature $T_{17}$. $\alpha^{*}$ is volume expansion coefficient. The value of it is $\alpha^{*}=1/T_{\mathrm{ave}}$ when air is regarded as an ideal gas. C and n are constant in Equation (10):

(12) $$C=0.59,n=0.25\;Gr\leq 3\times 10^{9}$$

(13) $$C=0.0292,n=0.39\;3\times 10^{9}\leq Gr\leq 2\times 10^{10}$$

(14) $$C=0.11,n=0.33\;Gr\geq 2\times 10^{10}$$

The convective heat transfer coefficient h* outside the insulation cotton with air at the measured section is:

(15) $$h^{*}=Nu\lambda /l_{\mathrm{ave}}$$

The heat loss outside the insulated cotton with air at the measured section is:

(16) $$Q_{\mathrm{loss}}=h^{*}l_{\mathrm{ave}}(w_{v}+2s_{2})\Delta T$$

The values of thermocouples 17–22 and the calculated heat loss in every corresponding surface for the copper oxide MGFHP filled with deionized water under the input heat of 24 W are shown in Table 3.

According to Table 3, the total heat loss for the thermocouples 18–22 corresponding surfaces is 0.474 W, accounting for 2.0% of the input heat. Additionally, the heat loss in every case is less than 2.6%. Hence, the heat transfer efficiency of the MGFHP is high, so that the heat loss can be neglected in the experiment.

Measurement accuracy was employed to evaluate the dependability of the measured parameters [25,26]. The relative uncertainty of thermal resistance $D_{T}$ is:

(17) $$D_{T}=\frac{S_{T}}{T}=0.5\%$$

As the input heat of the MFHP is from the ceramic heat chip, it can be obtained by the voltage *U* multiplying the current *I*. Voltage *U* was measured by the voltmeter with the relative uncertainty of 1.0%. Additionally, current *I* was measured by the ammeter with the relative uncertainty of 2.0%. Thus, the relative uncertainty of input heat $D_{Q}$ is:

(18) $$D_{Q}=\frac{S_{Q}}{Q}=\sqrt{{\left(\frac{S_{U}}{U}\right)}^{2}+{\left(\frac{S_{I}}{I}\right)}^{2}}\times 100\%=2.2\%$$

The relative uncertainty of thermal resistance $D_{R}$ is:

(19) $$D_{R}=\frac{S_{R}}{R}=\sqrt{{\left(\frac{S_{Q}}{Q}\right)}^{2}+D_{Q_{\mathrm{loss}}}^{2}+{\left(\frac{S_{T_{\mathrm{eva}}{-T}_{\mathrm{con}}}}{T_{\mathrm{eva}}-T_{\mathrm{con}}}\right)}^{2}}\times 100\%=6.7\%$$

The relative uncertainties of measured parameters are shown to be acceptable. The measured parameters can be used to analyze the thermal performance of the MGFHP.

## 3. Results and Discussion

### 3.1. Visualization Experiment

**Figure 6:**
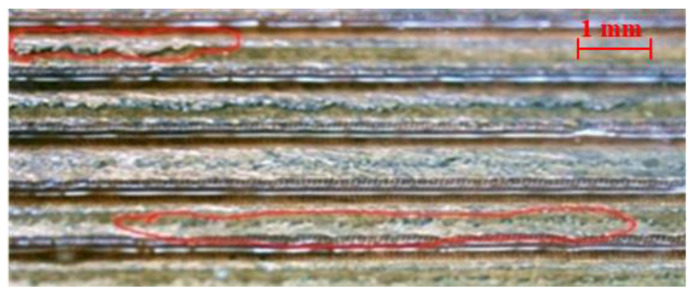
Water condensates on the lug boss between grooves under 8 W.

The working fluid of deionized water, anhydrous ethanol, or hexane is filled into the MGFHP, respectively. The distribution and flow process of liquid and vapor can be observed from the upper glass cover of the MGFHP. It is observed that when deionized water is injected into the MGFHP, liquid remains at the corner of the MGFHP. Additionally, deionized water still stays at the corner when external heat transfers into the MGFHP, which decreases the liquid amount in the grooves and lowers the working fluid distribution uniformity inside the MGFHP. As a result, all the grooves are not well moistened, causing the MGFHP to dry out easily. Moreover, a certain amount of liquid condensates on the lug boss between grooves at the condensation section rather than in the grooves of the MGFHP filled with deionized water, as shown in Figure 6. By contrast, liquid distributes evenly in grooves when anhydrous ethanol or hexane is injected into the MGFHP. This may be mainly caused by two reasons. On the one hand, the surface tension of water is distinctly higher than those of anhydrous ethanol and hexane. Anhydrous ethanol and hexane are more hydrophilic with copper, while deionized water possesses comparatively poorer wettability with copper [27,28]. On the other hand, the height of the vapor chamber is narrow, which gives rise to the capillary phenomenon at the corner of the MGFHP. It is easier to cause the uneven distribution of the working fluid in the MGFHP. Increasing the wettability of water with copper by oxidizing the grooves surface as CuO is found to contribute to uniformizing water distribution in grooves.

**Figure 7:**
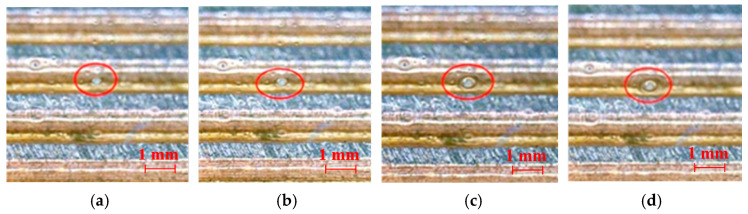
Boiling process of the MGFHP filled with anhydrous ethanol under 12 W: (**a**) 1 s; (**b**) 2 s; (**c**) 3 s; and (**d**) 4 s.

When the input heat is beyond a certain value, the boiling phenomenon is observed at the evaporation section of the anhydrous ethanol or hexane filled MGFHP. Figure 7 displays the boiling phenomenon when the MGFHP is filled with anhydrous ethanol under the input heat of 12 W. A bubble is shown to generate from the groove corner, which takes a total of 4 s. The boiling process of bubble includes generation, growth, departure, and arrival to the upper wall. Nucleate boiling at the initial state is found to enlarge the phase change area, taking away the heat and wetting the dried boss of groove wick, which can improve the thermal performance of the MGFHP. However, in the deionized water filled MGFHP, the nucleate boiling phenomenon is not observed. This may be due to the higher surface tension of deionized water that requires larger superheat and more significant driving power to invoke nucleate boiling, based on the heterogeneous nucleation theory [29].

**Figure 8:**
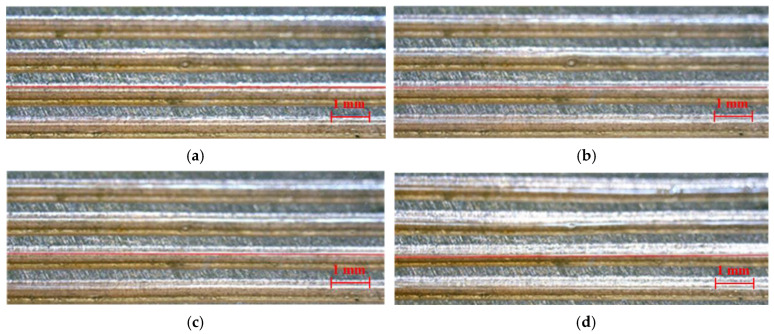
Formation of slope liquid level of the MGFHP filled with anhydrous ethanol under 12 W: (**a**) 1 s; (**b**) 80 s; (**c**) 160 s; and (**d**) 240 s.

Simultaneously, the liquid level is shown to continually decrease in the grooves of the MGFHP, especially at the evaporation section. Slope-shaped liquid level is formed along the grooves from the condensation section to the evaporation section. The liquid amount continuously reduces from the condensation section to the evaporation section. Working fluid evaporates at the evaporation section and condensates at the condensation section. Additionally, the inclination of the slope-shaped liquid level is more obvious with higher input heat, which is in accordance with our previous mathematical analysis [30]. Figure 8 presents the formation of slope-shaped liquid level for the anhydrous ethanol filled MGFHP under the input heat of 12 W. The stable slope-shaped liquid level is shown to form after the first 240 s.

### 3.2. Comparison of the Heat Transfer Performance between the MGFHP and Copper Plate

**Figure 9:**
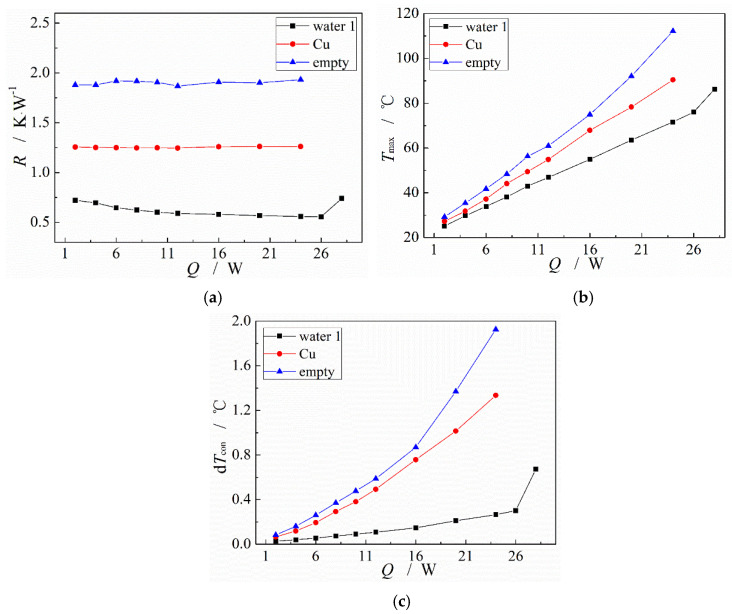
Comparison of the heat transfer performance between the MGFHP and copper plate: (**a**) thermal resistance; (**b**) maximum temperature at evaporation section; and (**c**) temperature nonuniformity at condensation section.

The heat transfer performance of MGFHP is compared with that of the pure copper plate with the same size. Figure 9 displays the thermal resistance, maximum temperature at the evaporation section, and temperature nonuniformity at the condensation section of pure copper plate, as well as empty MGFHP without any working fluid filling and MGFHP filled with deionized water as working fluid under different input heat amounts. Oxidation treatment was performed for the copper wick surface of the MGFHP filled with deionized water. Additionally, the filling ratio of deionized water is 1.0.

Figure 9 illustrates that the MGFHPs filled with deionized water show more excellent heat transfer performance than pure copper plate and empty MGFHP without any working fluid filling, possessing lower thermal resistance, lower maximum temperature at the evaporation section, and lower temperature nonuniformity at the condensation section, which is in accordance with the result from reference [31]. Under the input heat of 24 W, thermal resistance, maximum temperature, and temperature nonuniformity at the condensation section of the deionized water-filled MGFHP are almost lower than those of pure copper plate by 55.7%, 18.8 °C, and 1.07 °C, respectively. Additionally, they are almost lower than those of the empty MGFHP by 71.1%, 40.5 °C, and 1.67 °C, respectively. Working fluid plays a crucial role in the MGFHP to transfer the great heat. Thus, MGFHP filled with working fluid can be employed to cool electronic devices to avoid hot spots, lessen thermal stress, and expel great heat from the electronics effectively. Furthermore, the type and properties of working fluid need to be further studied.

### 3.3. Influence of Working Fluid Type and Wettability on the MGFHP Performance

**Figure 10:**
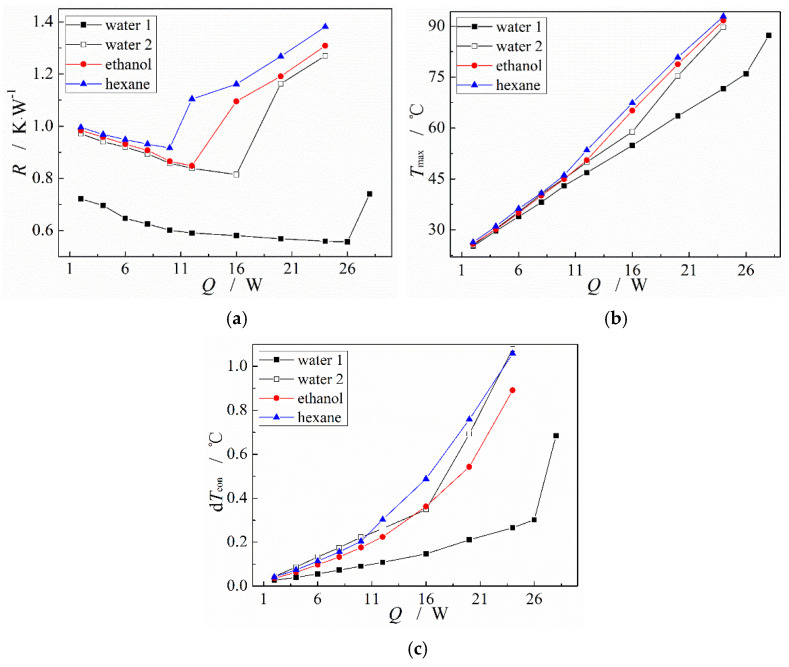
Heat transfer performance of the MGFHP under different input heat amounts with different working fluids: (**a**) thermal resistance; (**b**) maximum temperature at evaporation section; and (**c**) temperature nonuniformity at condensation section.

Three common and relative environment-friendly working fluids, deionized water, anhydrous ethanol, and hexane, are selected to investigate the heat transfer performance of the MGFHP with different working fluids. Figure 10 shows the thermal resistance, the maximum temperature at the evaporation section, and the temperature nonuniformity at the condensation section of the MGFHP filled with deionized water, anhydrous ethanol, or hexane under different input heat amounts. In the figure, water 1 represents the deionized water filled in the MGFHP with the oxidation treatment of copper wick surface, while water 2 denotes the deionized water filled in the MGFHP without the oxidation treatment of copper wick surface. Additionally, the filling ratio of working fluid in every case is 1.0.

Figure 10 shows that, when the input heat amount of the MGFHP filled with deionized water of water 1 or water 2, anhydrous ethanol or hexane exceeds 24 W, 16 W, 12 W, and 10 W separately, the thermal resistance, maximum temperature, and temperature nonuniformity of the MGFHP rise sharply. It indicates that the MGFHP filled with deionized water of water 1 or water 2, anhydrous ethanol, or hexane reaches the heat transfer limit under the input heat of 24 W, 16 W, 12 W, and 10 W separately because of the capillary limit [32]. The capillary force cannot balance the flow resistances inside the MGFHP [33], causing the MGFHP failure. From Figure 10a, before the MGFHP reaches the heat transfer limit, with the increase in the input heat amount, the thermal resistance decreases a bit for all the cases. MGFHP possesses higher thermal conductivity under a relatively higher input heat.

**Figure 11:**
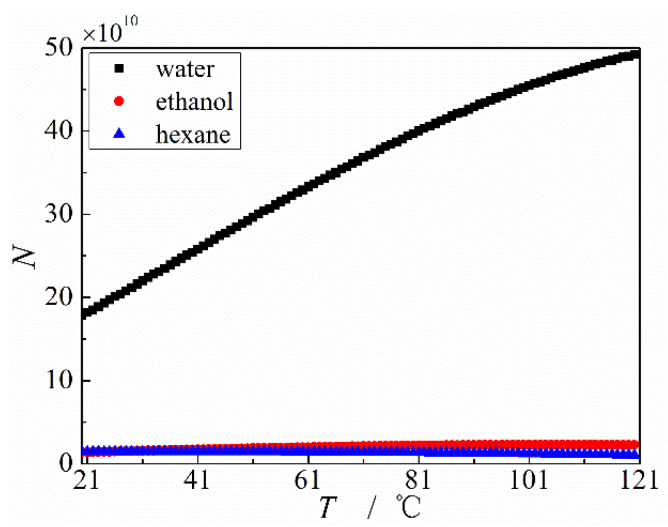
Merit factors of deionized water, anhydrous ethanol, and hexane.

Additionally, in comparison with MGFHP filled with anhydrous ethanol or hexane, the MGFHP filled with deionized water shows lower thermal resistance, lower maximum temperature, and higher heat transfer limit. Before the MGFHP reaches the heat transfer limit, thermal resistance, maximum temperature, and temperature nonuniformity at the condensation section of water 1 MGFHP are almost lower than those of the MGFHP filled with anhydrous ethanol or hexane by 31.1%, 3.7 °C, and 0.13 °C for the anhydrous ethanol-filled MGFHP and 34.4%, 3.1 °C, and 0.11 °C for the hexane-filled MGFHP, respectively. These phenomena are due to the more excellent physical properties of water than those of anhydrous ethanol and hexane. The physical parameters of latent heat, surface tension, specific heat, viscosity, density, and saturation pressure can obviously impact the performance of the MGFHP, including the flow velocity, pressure drop, evaporation and condensation of working fluid, the proportion of specific heat and latent heat in the heat transfer process, and the generation of bubble. In comparison with anhydrous ethanol and hexane, deionized water has higher surface tension to provide capillary force, larger liquid density to decrease fluid flow pressure drop, and higher latent heat to absorb/release heat at the evaporation/condensation section, which improves the heat transfer limit and enhances the heat transfer ability [34]. Merit factor $N=\sigma \cdot \rho_{l}\cdot h_{\mathrm{fg}}/\mu_{l}$ is frequently utilized to evaluate the influence of working fluid properties on the thermal performance of the MGFHP. The working fluid with high merit factor is beneficial for enhancing the thermal performance of the MGFHP. Merit factors of deionized water, anhydrous ethanol, and hexane are displayed in Figure 11. The merit factor of deionized water is shown to be particularly higher than that of anhydrous ethanol and hexane. Hence, deionized water presents better thermal performance than anhydrous ethanol and hexane.

In addition, Figure 10 shows that, for the copper MGFHP without oxidation treatment, the temperature distribution at the condensation section of the MGFHP filled with anhydrous ethanol or hexane is more uniform than that of the MGFHP filled with deionized water (water 2) before the MGFHP reaches the heat transfer limit. This is because the MGFHP drives the reflow of liquid chiefly by utilizing the meniscus at the narrow corner of grooves to offer capillary force. The phase change in liquid and vapor mainly occurs at the meniscus, so that the wettability of working fluid with groove is important. Anhydrous ethanol and hexane with higher wettability are more hydrophilic with original copper than deionized water with original copper. Condensed anhydrous ethanol and hexane spread evenly over the groove. Grooved wick with adequate working fluid is well wetted, which is beneficial for the working fluid to flow back from condensation section to evaporation section, leading to the uniform temperature distribution at the condensation section. On the contrary, deionized water cannot well wet the copper groove. Liquid droplet condensates on the boss of the grooved wick without entering the groove, which increases the local thermal resistance of the wick and reduces the circulation amount of the working fluid, causing the temperature distribution to be uneven inside the MGFHP. In view of the foregoing, the copper MGFHP filled with deionized water needs to be oxidized to increase its wettability. The oxidized MGFHP filled with deionized water (water 1) displays high wettability, which the contact angle of water with groove wick after oxidization decreases clearly from 63.7° to 13.5°. The heat transfer limit of the MGFHP after oxidization enlarges greatly from 16 W to 24 W. High wettability of working fluid with wick enhances the capillary force of wick, improving the heat transfer limit of the MGFHP. Additionally, the oxidized copper MGFHP filled with water displays lower thermal resistance, lower maximum temperature, and more uniform temperature distribution than the original copper MGFHP filled with water before reaching the heat transfer limit [35]. Thermal resistance, maximum temperature, and temperature nonuniformity at the condensation section of water 1 MGFHP are almost lower than those of water 2 MGFHP by 30.1%, 4.0 °C, and 0.21 °C, respectively, before the MGFHP reaches the heat transfer limit. Therefore, the wettability of working fluid with the wick can dramatically influence the performance of the MGFHP. Working fluid for the MGFHP needs to possess excellent surface tension, latent heat, wettability, and low viscosity. The temperature distribution of the MGFHP is more uniform with higher grooved wick wettability.

### 3.4. Influence of Working Fluid Filling Ratio on the MGFHP Performance

**Figure 12:**
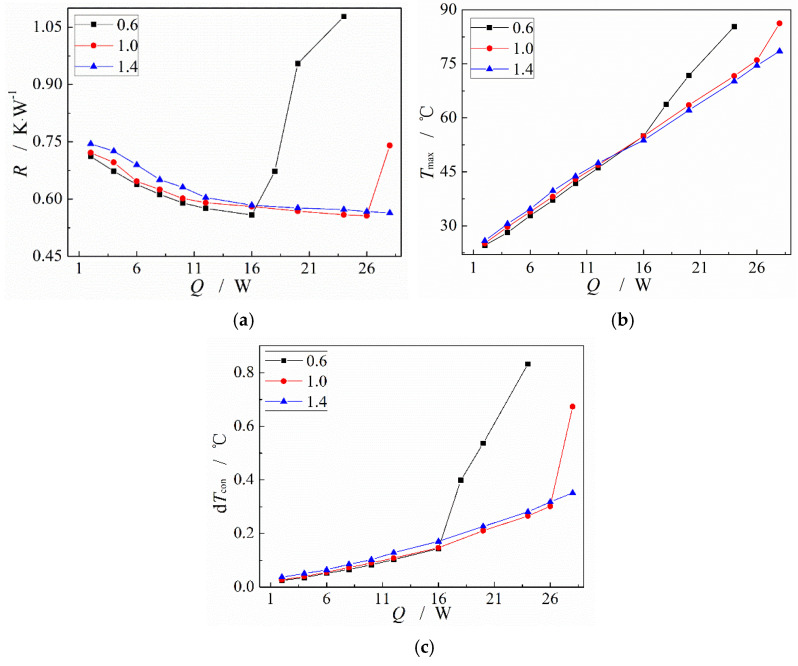
Heat transfer performance of copper oxide MGFHP filled with deionized water under different filling ratios: (**a**) thermal resistance; (**b**) maximum temperature at evaporation section; and (**c**) temperature uniformity at condensation section.

For the MGFHP mentioned in Section 3.3, the copper oxide MGFHP filled with water displays the best thermal characteristics due to the high transfer factor. In order to investigate the influence of working fluid filling ratio on the MGFHP performance, thermal resistance, maximum temperature at the evaporation section, and temperature nonuniformity at the condensation section of the copper oxide MGFHP filled with deionized water under different input heat amounts and working fluid filling ratios are depicted in Figure 12.

In Figure 12, under the condition of lower input heat amount, the MGFHP filled with deionized water with the filling ratio of 0.6 presents lower thermal resistance, lower maximum temperature, and lower temperature nonuniformity than that with the filling ratio of 1.0 or 1.4. However, when the input heat increases, the MGFHP filled with deionized water with the filling ratio of 1.0 or 1.4 shows more outstanding thermal performance. The deionized water-filled MGFHP with the filling ratio of 0.6 reaches the heat transfer limit when the input heat amount just exceeds 16 W. This is because, when the input heat is low, the liquid film in the grooved wick of the MGFHP is comparatively thick, which lowers the heat transfer rate of liquid film at the evaporation section. Especially for the MGFHP filled with a large quantity of working fluid, liquid may also block the cooling section. The blocked area does not participate in the working fluid circulation, which shortens the length of the condensation section. Heat needs to be transferred through the liquid inside the wick, but the thermal conductivity of liquid is much less than the latent heat from the phase change in the working fluid. Large thermal resistance exists in the MGFHP filled with high filling ratio working fluid. Thus, the MGFHP filled with deionized water with the filling ratio of 1.0 or 1.4 displays poorer heat transfer performance than that with the filling ratio of 0.6. However, with the increase in input heat, evaporation of working fluid accelerates, and more working fluid is required at the evaporation section. Liquid can flow back to the evaporation section in a timely manner for the MGFHP filled with sufficient working fluid, whereas the starting point of the liquid film regresses for the MGFHP filled with little amount of working fluid, which may cause the MGFHP to dry out easily [36].

## 4. Conclusions

The tendency of high heat flux and small space has been a major threat to the stable functioning of electronics. Mini-grooved flat heat pipe, with high compactness, excellent heat transfer ability, and uniform temperature distribution, can transfer substantial heat rapidly to the outside of the device and weaken the hot spot on the chips, which is suitable for cooling electronic devices under limited space. Visual and thermal experiments were conducted in this paper to determine the fluid flow and heat transfer characteristics of the MGFHP filled with different working fluids. The major results are obtained as follows:

(1) In the visual experiment, liquid level in slope shape is found in the MGFHP during working. Moreover, nucleate boiling phenomenon is observed in the MGFHP filled with anhydrous ethanol and hexane. Fluid flow and distribution are quite different in the MGFHP filled with different working fluids, which is affected by the physical properties of working fluid, surface state of grooved wick and limited working space.

(2) MGFHP filled with working fluid shows more excellent heat transfer performance than pure copper and empty MGFHP. Thermal resistance, maximum temperature, and temperature nonuniformity of the deionized water-filled MGFHP are almost lower than those of pure copper by 55.7%, 18.8 °C, and 1.07 °C under the input heat of 24 W, respectively. Moreover, they are lower than those of the empty MGFHP by 71.1%, 40.5 °C, and 1.67 °C, respectively.

(3) Thermal performance of the MGFHP filled with deionized water, anhydrous ethanol, or hexane is different. Heat transfer limits of MGFHPs filled with deionized water of water 1 and water 2, anhydrous ethanol, and hexane are about 24 W, 16 W, 12 W, and 10 W, respectively. Additionally, before the MGFHP reaches the heat transfer limit, thermal resistance, maximum temperature, and temperature nonuniformity at condensation section of water 1 MGFHP are almost lower than those of the MGFHP filled with anhydrous ethanol and hexane by 31.1%, 3.7 °C, and 0.13 °C for the anhydrous ethanol-filled MGFHP and 34.4%, 3.1 °C, and 0.11 °C for the hexane-filled MGFHP, respectively. The deionized water-filled MGFHP possesses lower thermal resistance and higher heat transfer limits than the anhydrous ethanol- or hexane-filled MGFHP, due to the excellent latent heat and surface tension of deionized water.

(4) The wettability of working fluid with wick affects the performance of the MGFHP significantly. Deionized water could gather in a certain place of the original copper MGFHP rather than evenly distribute inside the grooves, which undermines the temperature uniformity of the MGFHP. The copper oxide MGFHP filled with deionized water possesses high wettability, presenting better thermal characteristics, especially when the working fluid filling ratio is 1.0. Thermal resistance, maximum temperature, and temperature nonuniformity at the condensation section of copper oxide MGFHP filled with deionized water are almost lower than those of original copper MGFHP filled with deionized water by 30.1%, 4.0 °C, and 0.21 °C respectively, before the MGFHP reaches the heat transfer limit.

## Figures and Tables

**Table 1:** Geometric parameters of the MGFHP.

Parameter	*w*_v_ (mm)	*h*_v_ (mm)	*w*_g_ (mm)	*w*_b_ (mm)	*h*_g_ (mm)	*s*_1_ (mm)	*l*_eva_ (mm)	*l*_adia_ (mm)	*l*_con_ (mm)	*s*_2_ (mm)	*s*_3_ (mm)	*α* (°)
value	20.0	2.0	0.8	0.8	0.8	0.7	20.0	20.0	40.0	1.5	1.0	26.6

**Table 2:** Location and function of calibrated thermocouples.

Number	Location	Function
1–16	bottom surface of the MGFHP	calculate the heat transfer performance of the MGFHP
17	air temperature	measure the environment temperature
18–22	lower surfaces at evaporation, adiabatic, condensation sections, and upper surfaces of evaporation, adiabatic sections outside the insulation cotton of the MGFHP	calculate the heat loss in the MGFHP

**Table 3:** Location and function of calibrated thermocouples.

Thermocouple Number	17	18	19	20	21	22
Temperature (°C)	24.6	58.2	46.5	46.7	42.6	38.4
Corresponding Heat Loss (W)	/	0.148	0.087	0.088	0.068	0.083

## Data Availability

Some or all data used during the study are available from the corresponding author by request.

## Nomenclature

*D*	relative uncertainty
D*t*	temperature nonuniformity, °C
*Gr*	Grashof number
*H*	height, m
*h**	convective heat transfer coefficient, W·m^−2^·K^−1^
*h*_fg_	latent heat, J·kg^−1^
*l*	length, m
*N*	merit factor
*Nu*	Nusselt number
*Pr*	Prandtl number
*Q*	input heat, W
*Q*’	heat transfer amount, W
*R*	thermal resistance, K·W^−1^
*S*	uncertainty
*S*	thickness, m
*T*	temperature, °C
$\bar{T}$	average temperature, °C
*V*	volume, m^3^
*W*	width, m
**Greek symbols**	
*α*	half V-type angle value, °
*α**	volume expansion coefficient, K^−1^
*η*	filling ratio of working fluid
*λ*	thermal conductivity coefficient, W·m^−1^·K^−1^
*μ*	dynamic viscosity coefficient, N·s·m^−2^
ρ	density, kg·m^−3^
σ	surface tension, N·m^−1^
**Subscripts**	
adia	adiabatic
ave	average
b	boss
con	condensation
eva	evaporation
f	fluid
g	groove
I	current
I	order of number
L	liquid
Max	maximum
Q	input heat
Q_loss_	heat loss
R	thermal resistance
T	temperature
U	voltage
V	vapor
W	wall

## References

[1] Khalaj A.H., Halgamuge S.K. (2017). A review on efficient thermal management of air-and liquid-cooled data centers: From chip to the cooling system. Appl. Energy.

[2] Alijani H., Cetin B., Akkus Y., Dursunkaya Z. (2018). Effect of design and operating parameters on the thermal performance of aluminum flat grooved heat pipes. Appl. Therm. Eng..

[3] Jung E.G., Boo J.H. (2017). A numerical study on the heat and mass transfer of a micro heat pipe with a phase-change interface analysis. Heat Mass Transf..

[4] Qu J., Wu H., Cheng P., Wang Q., Sun Q. (2017). Recent advances in MEMs-based micro heat pipes. Int. J. Heat Mass Transf..

[5] Mihai I., Suciu C., Picus C.M. (2022). Considerations for the maximum heat load and its influence on temperature variation of the evaporator in flat MHPs in transient regimes. Micromachines.

[6] Li J., Lv L., Zhou G., Li X. (2019). Mechanism of a microscale flat plate heat pipe with extremely high nominal thermal conductivity for cooling high-end smartphone chips. Energy Convers. Manag..

[7] Liang K., Li Z., Chen M., Jiang H. (2019). Comparisons between heat pipe, thermoelectric system, and vapour compression refrigeration system for electronics cooling. Appl. Therm. Eng..

[8] Lu M.C. (2010). Exploring the Limits of Boiling and Evaporative Heat Transfer Using Micro/Nano Structures. Ph.D. Thesis.

[9] Lips S. (2018). Contribution to the Experimental and Theoretical Study of Phase-Change Heat Transfer for the Thermal Management of Electronics. Ph.D. Thesis.

[10] Morris S.J.S. (2003). The evaporating meniscus in a channel. J. Fluid Mech..

[11] Singh R., Akbarzadeh A., Mochizuki M. (2009). Effect of wick characteristics on the thermal performance of the miniature loop heat pipes. ASME J. Heat Transf..

[12] Tang H., Tang Y., Li J., Sun Y., Liang G., Peng R. (2018). Experimental investigation of the thermal performance of heat pipe with multi-heat source and double-end cooling. Appl. Therm. Eng..

[13] Alijani H., Cetin B., Akkus Y., Dursunkaya Z. (2019). Experimental thermal performance characterization of flat grooved heat pipes. Heat Transf. Eng..

[14] Lips S., Lefèvre F., Bonjour J. (2009). Nucleate boiling in a flat grooved heat pipe. Int. J. Therm. Sci..

[15] Wang C., Liu Z., Zhang G. (2013). Experimental investigations of flat plate heat pipes with interlaced narrow grooves or channels as capillary structure. Exp. Therm. Fluid Sci..

[16] Chen J.S., Chou J.H. (2014). Cooling performance of flat plate heat pipes with different liquid filling ratios. Int. J. Heat Mass Transf..

[17] Wang G., Quan Z., Zhao Y., Wang H. (2019). Performance of a flat-plate micro heat pipe at different filling ratios and working fluids. Appl. Therm. Eng..

[18] Saygan S., Akkus Y., Dursunkaya Z., Cetin B. (2022). Capillary boosting for enhanced heat pipe performance through bifurcation of grooves: Numerical assessment and experimental validation. Int. Commun. Heat Mass.

[19] Saygan S., Akkus Y., Dursunkaya Z., Cetin B. (2022). Fast and predictive heat pipe design and analysis toolbox: H-PAT. Isi Bilimi Ve Tek. Derg. J. Therm. Sci. Technol..

[20] Wong S.C., Chen C.W. (2012). Visualization and evaporator resistance measurement for a groove-wicked flat-plate heat pipe. Int. J. Heat Mass Transf..

[21] Tio K.K., Hung Y.M. (2015). Analysis of overloaded micro heat pipes: Effects of solid thermal conductivity. Int. J. Heat Mass Transf..

[22] Zhang W., Wen X., Yang S., Berta Y., Wang Z.L. (2003). Single-crystalline scroll-type nanotube arrays of copper hydroxide synthesized at room temperature. Adv. Mater..

[23] Iverson B.D., Davis T.W., Garimella S.V., North M.T., Kang S.S. (2007). Heat and mass transport in heat pipe wick structures. J. Thermophys. Heat Transf..

[24] Liu C., Chen Y., Feng D., Zhang H., Miao J., Feng Y., Yan Y., Zhang X. (2022). Experimental study on temperature uniformity and heat transfer performance of nitrogen loop heat pipe with large area and multi-heat source. Appl. Therm. Eng..

[25] Solomon A.B., Kumar A.M.R., Ramachandran K., Pillai B.C., Kumar C.S., Sharifpur M., Meyer J.P. (2017). Characterization of a grooved heat pipe with an anodised surface. Heat Mass Transf..

[26] Liao X., Jian Q., Zu S., Li D., Huang Z. (2021). Visualization study and analysis on the heat transfer performance of an ultra-thin flat-plate heat pipe. Int. Commun. Heat Mass.

[27] Faghri A. (1995). Heat Pipe Science and Technology.

[28] Rag R., Sobhan C. (2009). Computational analysis of fluid flow and heat transfer in wire-sandwiched microheat pipes. J. Thermophys. Heat Transf..

[29] Carey V.P. (2007). Liquid-Vapor Phase-Change Phenomena.

[30] Xin F., Ma T., Wang Q. (2018). Thermal performance analysis of flat heat pipe with graded mini-grooves wick. Appl. Energy.

[31] Oshman C., Shi B., Li C., Yang R., Lee Y.C., Peterson G.P., Bright V.M. (2011). The development of polymer-based flat heat pipes. J. Microelectromech. Syst..

[32] Jiao A.J., Ma H.B., Critser J.K. (2007). Evaporation heat transfer characteristics of a grooved heat pipe with micro-trapezoidal grooves. Int. J. Heat Mass Transf..

[33] Li J., Lv L. (2016). Experimental studies on a novel thin flat heat pipe heat spreader. Appl. Therm. Eng..

[34] Patankar G., Weibel J.A., Garimella S.V. (2017). Working-fluid selection for minimized thermal resistance in ultra-thin vapor chambers. Int. J. Heat Mass Transf..

[35] Peng Y., Liu W., Liu B., Liu J., Huang K., Wang L., Chen W. (2015). The performance of the novel vapor chamber based on the leaf vein system. Int. J. Heat Mass Transf..

[36] Rice J., Faghri A. Analysis of porous wick heat pipes, including capillary dry-out limitations. Proceedings of the ASME 2005 International Mechanical Engineering Congress and Exposition.

